# Systemic therapies for inflammatory eye disease: Past, Present and Future

**DOI:** 10.1186/1471-2415-13-18

**Published:** 2013-04-24

**Authors:** Alastair K Denniston, Andrew D Dick

**Affiliations:** 1 Ophthalmology Department, University Hospitals Birmingham NHS Trust, Queen Elizabeth Hospital Birmingham, Mindelsohn Way, Edgbaston, Birmingham, B15 2WB, UK; 2 Faculty Medicine and Dentistry Ophthalmology, University of Bristol, Bristol Eye Hospital, Lower Maudlin Street, Bristol, BS1 2LX, UK; 3 National Institute of Health Research Biomedical Centre, Moorfields Eye Hospital NHS Foundation Trust and UCL Institute of Ophthalmology, London, UK

## Abstract

In this review we consider the current evidence base for treatments in inflammatory eye disease, and in particular uveitis, from a historical perspective. We consider the challenges that have traditionally hindered progress in inflammatory eye disease including small target populations, heterogeneous disease groups, poorly defined phenotypes, diagnostic inconsistency, subjective outcome measures, specific issues around visual acuity as an outcome measure and low commercial interest. Strategies to address these issues are considered de novo and with reference to recent advances outside of ophthalmology and highlight the promise for ocular inflammation. Progress in these specialties has included the development of thriving clinical-trial cultures, public-private partnerships, pathogenetic- and structure-led drug design, efficient drug development pipelines, and biomarker-defined treatment protocols enabling personalization of medicine. Although there are challenges, these are exciting opportunities as we seek to develop safe and effective treatments for patients with inflammatory eye disease.

## Introduction

Philosophically we recognize that the past informs our present but realize less that our present actions and thoughts dictate our future. To this end, as we consider the progress that has been made in the systemic treatment of inflammatory eye disease, there is a danger that we over-value recent successes due to their proximity rather than merit. Newer drugs, like most recent news stories, may attract more of the reviewer’s attention: they are the recent arrivals, usually accompanied by a promise of delivering better outcomes for our patients and with the advantage of having had time to demonstrate their short-term efficacy but not their long-term consequences (whether good or ill). Objective assessment of the merits of these systemic therapies requires the high-quality evidence that comes from well-designed randomized clinical trials, with extended follow-up to ensure late effects are not missed. We are currently a long way from being in that position. In the clinic we are inured to this and, on the basis of the limited available evidence alongside experience and anecdote [1], we make the best therapeutic choice we can. We are reassured because cohort studies infer that a majority of patients improve [2-8], but we do not know whether to what extent a placebo or indeed no treatment would have had the same effect. This is clearly an unsatisfactory position and we need to move on.

In this review we have not sought to appraise the individual systemic therapies currently available to us; this is important but is covered in detail elsewhere [9,10]. Rather we seek to provide a critical assessment of the state of the field: where we started from, what progress has been made and a vision of how we can bridge the considerable gap to get to where we need to be – providing therapies which are proven to be effective and safe and targeted to the individual patient in front of us.

## Looking back: dark ages to the renaissance

A characteristic of our medical past was that, though well-motivated by a desire to provide better care to our patients, actual therapeutic practice was often driven more by personality and confidence rather than by careful study and appraisal. Thus up to the early 20th century the therapeutic armory of the physician included useless or harmful practices such as bleeding, purging, and widespread provision of tonics, mercuric compounds and hypnotics. Patients with ocular inflammation were similarly provided for. In the 18th century a popular propriety remedy ‘Golden Eye Ointment’ was a mercury oxide that was normally mixed with hog’s lard, and was promoted as being useful for ‘all forms of chronic ophthalmia’. In the late 19th century, Savory's Compendium of Domestic Medicine recommended “In inflammation of the eye, originating from cold or accident, it is advisable to apply three or four leeches round the orbit… they will always be found to be safe, and generally a successful remedy [11].” Rather more useful was the application of tincture of belladonna to induce pupil dilation. In the 1930s the advent of commercially available antibiotics heralded the start of rational treatment for infectious eye disease, but also gave some indication that much inflammatory eye disease was actually non-infectious in origin. It was recognized then that non-infectious inflammatory eye disease would require an entirely different therapeutic approach.

In 1950 the use of corticosteroids for chronic anterior uveitis by Gordon and McLean signaled a major break-through in the management of inflammatory eye disease [12]. With high efficacy and fast onset of action, corticosteroids quickly established their role in the acute treatment of inflammatory disease (i.e. induction of remission) but their wide-ranging systemic side-effects have always limited their long-term usage (i.e. for maintenance of remission). The latter half of the 20th century saw the introduction of immunosuppressants such as the alkylating agents (cyclophosphamide [13,14] and chlorambucil), the antiproliferative agents (including methotrexate [15], azathioprine [16] and mycophenolate [17]) and the calcineurin antagonists (notably ciclosporin [18] and tacrolimus [19]). This was a revolution in our approach to therapeutics, representing a new paradigm of understanding immune mechanisms in animal models, using these animal models as a translational path to provide evidence to move into man and finally conducting early proof of concept studies [18,20-24]. Although slower in onset and requiring more intensive monitoring than corticosteroids, these agents were generally more suitable for maintenance therapy due to their improved systemic safety profile, providing that they were monitored appropriately [17,19,25]. However, these agents, like corticosteroids, lack specificity. They cause a generalized immunosuppression which renders the patient more vulnerable to opportunistic and severe infection. Although some success has been achieved, a more targeted approach was sought.

The advent of biologic medical products (‘biologics’) has been a major landmark in the treatment of inflammatory disease. Drugs such as the anti-TNF agents (e.g. etanercept, infliximab, adalimumab) and the anti-CD20 agent rituximab have already revolutionized the management of many rheumatic diseases, and the impact is now being realized within ophthalmology. The development of a biologic should arise out of a knowledge of the pathogenesis of the inflammatory disease, and be targeted to inhibit or modulate specific components. This approach has been taken for uveitis with the potential to be more effective in controlling disease with less impact on the patient’s overall health. Leading the charge within ophthalmology are the anti-TNF therapies. We among others have undertaken a program of work which has investigated the role of TNF-α in the pathogenesis of uveitis [26], leading to the development of an anti-TNF-α fusion protein (TNFr-Ig) in 1996 which we evaluated first in an animal model of uveitis [27,28], and subsequently in a Phase II/proof of concept clinical trial in 2004 [4,29]. In this study, 71% of the patients achieved complete cessation of intraocular inflammation following TNFr-Ig therapy and a reduction in concomitant immunosuppression was possible in 65% of cases [29]. Importantly TNFr-Ig therapy appeared to be effective across uveitic subtypes, including Behçet’s disease, idiopathic intermediate uveitis, multifocal choroiditis and sympathetic ophthalmia. These findings are supported by a large body of work from other groups [30-35], and subsequent reviews and editorials have highlighted the impact of anti-TNF-α therapies in ophthalmic practice [7,36-39].

These are exciting times with an ever-expanding range of immunosuppressants on the FDA-approved list. But the question remains: how much progress have we actually made? Put another way, when faced with a patient with sight-threatening inflammatory eye disease, do we actually know which drug to use?

Most of the immunosuppressants used in ocular inflammatory disease were originally developed for use in transplant medicine, rheumatic disease or other systemic inflammatory diseases. Very few have high level evidence for their use in ocular inflammation, and almost all are used off-label. A recent study which surveyed uveitis experts’ approach to a number of clinical scenarios, found that whilst there was a general consensus on the overall approach to immunosuppression (i.e. starting with corticosteroids first line, and subsequent initiation of a steroid-sparing immunosuppressant) there was considerable variation in which second line agent to use and at what dose. This is perhaps unsurprising since the study also reported that these experts suggested the evidence underlying their decisions was either absent or relatively weak (evidence levels III or IV based on the evidential hierarchy described by the United States Agency for Health Care Policy and Research) [40], and that in most cases personal experience was a key factor in their decision-making [1]. In this regard we should not under-estimate recent progress which has seen an increasing number of prospective studies on standard and novel therapies [41-45], and larger standardized retrospective studies [2-8]. A balanced approach is needed. Our current shortage of large-scale randomized controlled trials should not lead to us ignore the evidence that is already available for therapeutic efficacy and safety. Conversely we must recognize the ‘evidence-gap’ that remains and identify strategies that will enable us to bridge it.

There are a number of reasons we lag behind other specialties, notably our small target population, heterogeneous disease groups, poorly defined phenotypes, uncertain pathogenesis, diagnostic inconsistency, subjective outcome measures, visual acuity issues, and until recently little commercial interest and a non-trial culture. These are some of the factors that need to be addressed if we are to conduct the trials and build the evidence base which will enable us to make informed therapeutic decisions in the future.

## Reflecting on now: the Age of enlightenment

In most ‘inflammologies’ (rheumatology, renal medicine and the rest) the last decade has been characterized by major multicentre studies that have established the efficacy and safety of established or novel therapies in a randomized controlled format (vs. placebo or standard care). In inflammatory eye disease we have performed very few such studies, but encouragingly there has been a major effort to address the blocks that have hitherto limited our progress. A striking characteristic of this new phase is the extent to which the whole inflammatory eye disease community is working together with increasing international and cross-specialty collaboration. As we consider these issues below we have focused on uveitis, but the arguments apply equally well to most other forms of inflammatory eye disease (such as ocular mucous membrane pemphigoid, autoimmune keratitis, scleritis, myositis, inflammatory orbitopathies and inflammatory neuropathies):

### Problem 1: small target population

Uveitis has an annual incidence of 14–50 per 100 000 [46], with a prevalence of around 38–115 per 100 000 in the general population [47-49]. Since the majority of these cases are acute anterior uveitis responsive to topical therapy, the population of patients who need systemic therapy (and are eligible for clinical trials of immunosuppressants) is small. Other inflammatory diseases such as scleritis have a higher proportion requiring systemic therapy, but are overall much less common. It is partly in response to this, that increasing collaboration has emerged in recent years. One example is the SITE consortium, a collaboration of five academic ocular inflammation practices in the United States who undertook a retrospective cohort study primarily to investigate long-term adverse events occurring in patients on systemic therapies for ocular inflammatory disease; although not the primary aim, this study also provides interesting data on outcomes in the use of these drugs [50]. The study group comprised 7957 (of whom 2340 were treated with systemic immunosuppression) patients observed over 68751 visits, spanning 14.910 person years [2-6]. Whilst accepting the limitations inherent in a retrospective study, this is an important milestone in being the largest dataset of immunosuppression in ocular inflammatory disease.

### Problem 2: heterogeneous disease groups

Ocular inflammatory disease is very heterogeneous group. Within uveitis, classification is generally by anatomical grouping and etiology, including the presence or absence of systemic disease. ‘Splitting’ i.e. defining a very pure cohort (e.g. Posterior uveitis of Birdshot pattern) leads to a very small target population, whereas ‘lumping’ enables easier recruitment but may be clinically meaningless due to the range of disease included. This is true both for routine clinical practice and for clinical trials. In clinical trials maximization of the signal: noise ratio is critical. An intervention may be highly effective for a disease but will fail to return a statistically significant benefit if it is trialed on too broad a group of patients (e.g. patients who have conditions that look superficially similar but differ fundamentally in etiology).

### Problem 3: poorly defined phenotypes

In addition to its heterogeneity as a group, the uveitis subtypes are imperfectly defined with no diagnostic criteria with high enough sensitivity and specificity to reliably separate all cases of one uveitis entity from another. This leads to considerable variation in classification and diagnosis. To some extent such markers may only become apparent as we better understand disease etiology. This has occurred in the field of retinal dystrophies where there has been a shift from grouping by somewhat arbitrary pictorial descriptions (e.g. butterfly-shaped macular dystrophy) to defining disease by the gene responsible (e.g. a peripherin/RDS retinal dystrophy). This is important not only because it leads to a more clear-cut and objective classification of disease, but also because a classification based on real differences in etiology is more likely to translate into an appropriately targeted therapeutic approach.

### Problem 4: diagnostic inconsistency

Within uveitis the definition of syndromes by pattern and the lack of clear diagnostic markers leads to significant variation between clinicians in the classification of uveitis entities. Currently a major international collaboration - the Standardization of Uveitis Nomenclature consortium - is seeking to define classification criteria for 28 different uveitis syndromes [51]. The consortium have identified 194 uveitic terms and ‘dimensions’ including 87 unique terms that are specifically used by uveitis specialists to describe signs and symptoms. These were mapped to the 28 uveitic syndromes under consideration. Currently 250 cases per uveitis syndrome are being gathered which will be used to validate the mappings and to form the basis of a classification criteria and a proposed ontology for uveitis [52]. Although this system is based on pattern rather pathogenesis, this project does have the potential to significantly improve standardization of uveitis classification with benefits for both clinical practice and trials.

### Problem 5: subjective outcome measures

One of the advantages of managing inflammation in the eye (as opposed to elsewhere in the body) is that the transparent nature of the cornea and visual axis enables us to see and score inflammatory activity and titrate treatment accordingly. The flip-side is that we have accepted these same subjective activity indices in clinical trials. Non-invasive technologies which can objectively quantify the key parameters of cellular activity, flare and vitreous haze are needed. With regard to the assessment of AC flare, laser flare meters have potential but current models are relatively time-consuming and cumbersome, with some concerns over variability especially in the non-ideal patient (e.g. posterior synechiae, patient mobility). Some models have sought to also quantify cellular activity but with limited success. With regard to the posterior segment the NEI vitreous haze score described by Nussenblatt *et al.* (which depends on the clinician scoring the clarity of the optic disc which can be compared to a standard set of photographs) is the key outcome recognized by the FDA [53]. Although it is the gold-standard, it has a number of limitations including that it is (1) subjective [54]; (2) non-continuous, leading to very large steps in disease activity between categories; (3) poorly discriminatory at lower levels of vitreous haze, with most cases of active uveitis being scored at 0.5+ or 1+; and (4) limiting of recruitment and sensitivity in a clinical trial context (where a 2 point change is usually required to be counted as a significant change).

An adaptation of this technique proposed by Davis and colleagues is to score clarity on photographs (rather than on the live biomicroscopic view of the NEI technique) which can then be compared to a more extensive set of reference images leading to a potentially more discriminatory 0–9 score (vs. the 0, 0.5, 1, 2, 3 and 4 scale of the SUN modification of the Nussenblatt) [55]. Overall however this is still a subjective technique and shares most of the key limitations of the standard vitreous haze score. A truly objective measure is likely to be based on quantification of vitreous density or reflectivity using current or future imaging technologies.

### Problem 6: the ‘distraction’ of visual acuity

Whilst it is absolutely right that our key aim is to retain and restore vision, we must recognize that visual acuity is a very poor marker of the efficacy of a drug in inflammatory eye disease. The impact of uveitis on visual acuity will depend on both the activity of the disease and the damage caused by the disease. Thus whilst vision may improve due to treatment-induced improvement in cystoid macular edema, vitritis, keratic precipitates and aqueous clarity, this benefit may be obscured by loss of vision due to cataract, band keratopathy, macular scarring or even glaucoma. A further complexity arises in that some of these types of damage are reversible (notably cataract and band keratopathy) leading to the slightly bizarre scenario that their correction has to be specifically prohibited within most clinical trials. Additionally it is impossible to accurately quantify the extent to which each factor is contributing to the reduced visual acuity. Although the clinician is used to making these judgment calls every day in clinic - for example when deciding the extent to which advancing cataract or persistent cystoid macular edema is responsible for a fall in vision – these estimates are very approximate and frequently shown to be incorrect. It should also be recognized that visual acuity is a subjective parameter which despite every effort towards standardization may be affected by factors such as patient mood, general health and compliance. Visual outcome remains the core purpose for clinical trials in inflammatory eye disease, but it is less clear how it should be utilized as a trial endpoint.

The increasing and important focus on patient reported outcome measures (PROMs) as a component of clinical trials may start to capture some of these aspects of the overall benefit experienced by the patient. It is encouraging that more recent clinical trials in uveitis have often incorporated measures both of quality of life and health utility. These include specific vision-related quality of life measures (such as the 25-item NEI-Visual Function Questionnaire, NEI-VFQ25 or the Vision-related Quality of Life Core Measure, VCM-1) [56-61] general health-related quality of life measure (such as the short form-36, SF-36) [58,59,61,62] and health utility measures (such as the EuroQol 5-dimension, EQ-5D, and Visual Analogue Scores) [61,63].

### Problem 7: Low commercial interest

In the past pharmaceutical companies have shown little interest in ocular inflammatory disease, partly due to the relatively small market. Encouragingly the last decade has seen a surge of interest with industry-sponsored studies of both novel agents – such as the calcineurin antagonist voclosporin and the anti-IL17 agent secukinumab – and of novel routes for older drugs (e.g. intravitreal corticosteroid implants, intravitreal sirolimus) and providing ultimately robust evidence of anti-TNF blockade.

## Looking forward: industrial revolution to information Age

Addressing the challenges outlined above will enable us to design and conduct the clinical trials that will be able to inform our therapeutic decisions in inflammatory eye disease. In the meantime the bar is being raised. In this last section we look at a couple of case-studies that highlight some of the exciting developments that are leading to major advances in other specialties.

### Ivacaftor: detailed understanding of pathogenesis leads to specific drug-design matched to carefully defined disease groups

Cystic Fibrosis is a life-limiting multisystem disorder arising from abnormal function or complete loss of function of the protein, Cystic Fibrosis Transmembrane Conductance Regulator (CFTR). The CFTR is an ABC transporter-class ion channel in epithelial cell membranes which regulates the constituents of sweat, mucus and digestive fluids (reviewed [64]). Although non-specialists often regard CF as a single condition, there is significant variation in the severity and organ systems preferentially affected. Since identification of the CFTR gene in 1989, it has become increasingly clear that a key part of this variation in phenotype is dependent on the specific mutation in the CFTR gene. G551D is a mutation which affects around 5% of CF patients. Critically, although the G551D mutation does cause impaired ion transport of the CFTR, the CFTR protein is still expressed on the surface of the epithelial cell [65]. It was therefore postulated that it might be possible to intervene in this subgroup of CF patients by using drugs that potentiate the existing CFTR function. Increasing interest led to the Cystic Fibrosis Foundation contributing an estimated $75 million to support the development of one such drug Ivacaftor (Kalydeco) by Vertex Pharmaceuticals. In 2011 two placebo-controlled clinical studies comprising a total of 213 patients showed significant and sustained improvement in lung function [66]. In early 2012 Ivacaftor was approved by the FDA for use in patients with the G551D mutation. Thus for the first time there is a therapy which actually targets and restores function within the abnormal protein in CF, albeit in a carefully defined subgroup of the disease and with an estimated annual cost of $294, 000 per patient.

Although posterior segment uveitis is probably more heterogeneous than CF (at least in terms of etiology and pathogenesis), one of the key lessons here is that the development of drugs such as Ivacaftor is only possible when there is accurate classification of subtypes of disease based on real differences at the pathogenic level. Within ophthalmology we see this approach showing promise in the field of retinal dystrophies where accurate definition of genotype opens the possibility of targeted gene therapy [67]. As our understanding of the pathogenesis of the ocular inflammatory diseases improves, therapeutically useful classification of disease should follow.

### Vemurafenib: established drug-development pipeline enables rapid translation for patient benefit

Metastatic melanoma has a very poor prognosis with a median survival of 8 to 18 months after diagnosis for stage IV melanoma. In 2002 Davis et al. noted that around half of melanomas have an activating mutation in the B-RAF oncogene, a regulator of the MAP kinase pathway that controls cell proliferation [68]. Subsequently it was shown that 90% of these mutations are of the V600E type. In 2008 Tsai et al. published their results documenting the targeted development of a specific inhibitor of the mutant V600E BRAF and its effects on both melanoma cell lines and tumor xenograft models [69]. In 2010 the first Phase I trial defined the maximal tolerated dose and noted frequent tumor responses [70]. In 2011 the first phase 2 and first phase 3 reported efficacy data, the latter demonstrating significant benefits with a six month survival of 84% (vs. 64% for standard treatment) and a 74% relative reduction in death or disease progression compared to standard treatment [71,72]. Later that same year the drug was approved by the FDA for the treatment of late stage melanoma, a development time of just nine years since the original Davis paper.

Within uveitis, the recent development of the anti-IL17 therapy secukinumab and rapid translation into clinical trials for non-infectious posterior segment disease is an encouraging example of how ocular inflammatory disease can start to benefit from established drug development pipelines [73]. Although it is too early to comment on whether secukinumab will be a major chapter or only a foot-note in the management of uveitis, its targeted development from animal model to clinical trial has been a significant milestone within the field.

### I-SPY-2: adaptive trial design and public-private partnership underpin innovative rolling drug-testing program

I-SPY-2 is a phase II rolling drug screening program to test new therapies for breast cancer [74]. The design has six treatment arms, including an arm for standard therapy. A key feature is that randomization is adaptive (within biomarker subsets) whereby the probability of being assigned to a particular treatment arm increases if the outcome of the prior patients in the group was good [75,76]. As the trial evolves the treatment arms are replaced either because they show sufficient success to graduate to a smaller, focused phase III study or because they are terminated due to lack of benefit (based on Bayesian predictions of success or futility). This design means that the more successful a drug, the faster that it will move through the screening process. It also means that trial participants will tend to receive the more effective treatments. Fewer patients are required (per drug outcome) and it is predicted to be significantly cheaper than standard single drug non-adaptive designs. Up to 12 drugs will be tested through the I-SPY-2 program. This study is a collaboration across the USA between the National Cancer Institute, the FDA and around 20 major cancer centers, with active engagement by pharmaceutical companies who are providing their drugs for free. There is a commitment to publish all key trial results and the data arising from the trial will be managed by the independent non-profit organization the Foundation for the NIH. It is probably too early to judge but if successful, I-SPY-2 could revolutionize our approach to clinical trials [75,76].

## Conclusions: A Bright Future

This ‘alternative’ universe may currently seem a long way off: a vibrant clinical-trial culture, public-private partnerships, pathogenetic- and structure-led drug design, efficient drug development pipelines, biomarker-defined treatment protocols enabling personalization of medicine, and a bench-to-approval transit time of less than ten years. The successes in other specialties show that this is achievable. Reflecting on how far we have to go helps set our priorities and targets. Progress is being made, and it is our hope that these advances in pathogenesis, disease classification, methodology and trial design supported by a new culture of collaboration will finally enable us to treat patients with inflammatory eye disease with systemic therapies that are effective, safe … and proven.

## Competing interests

AKD declares that he has no competing interests. ADD is on the advisory board for Novartis.

## Authors’ contributions

AKD and ADD jointly contributed to the writing of the manuscript, and both read and approved the final manuscript.

## Pre-publication history

The pre-publication history for this paper can be accessed here:

http://www.biomedcentral.com/1471-2415/13/18/prepub
